# Dental caries misinformation on Instagram: Investigation of sharing and engagement factors

**DOI:** 10.1590/1807-3107bor-2026.vol40.036

**Published:** 2026-06-22

**Authors:** Tamires de Sá Menezes, Ana Maria Jucá, Matheus Lotto, Agnes Cruvinel, Thiago Cruvinel

**Affiliations:** (a) Universidade de São Paulo – USP, Bauru School of Dentistry, Department of Pediatric Dentistry, Orthodontics, and Public Health, Bauru, SP, Brazil.; (b) Universidade de São Paulo – USP, Bauru School of Medicine, Bauru, SP, Brazil.

**Keywords:** Communication, Infodemiology, Oral Health, Social Media

## Abstract

This study aimed to identify misinformation related to dental caries in Brazilian Portuguese on Instagram, analyzing factors associated with its dissemination and corresponding engagement metrics. Two independent investigators conducted a qualitative analysis of 500 posts published between August 2018 and August 2022. Posts were selected using the CrowdTangle tool and assessed for author's profile, content, motivation, facticity, format, and sentiment. Data were evaluated using descriptive analysis, the Mann–Whitney U test, and path analysis through generalized structural equation modeling. Topic modeling analysis was also performed using QDA Miner software with the WordStat module to identify underlying topics in dental caries-related information and misinformation. The findings indicated that 21.8% of the analyzed posts contained misinformation, primarily on content shared by regular users regarding dental caries treatment. No significant relationship was observed between misinformation and user engagement; however, posts with misinformation showed a significantly higher median total interaction than those without. Overperformance scores were positively correlated with business and health-related profiles, as well as higher total interaction levels. Financial motivation was less frequently associated with treatment-related posts, reflecting the tendency of promotional content to focus on preventive technologies. Lastly, whereas the misinformation predominantly addressed dental caries treatment, diet, microorganisms, and oral hygiene, the accurate information mainly covered prevention, treatment, and oral hygiene. Although these findings were not associated with higher user engagement, they underscore concerns regarding the dissemination of misleading information about dental issues online. Business and health professional profiles demonstrated good potential to disseminate reliable information with higher engagement metrics.

## Introduction

The digital revolution in recent decades has increased access to information, including in the field of health.^1,2^ This phenomenon has triggered a new paradigm in which the proliferation of health information through social media has raised significant concerns regarding the prevalence of false, inaccurate, and misleading information and its impact on decision-making and individual beliefs.^1^

This shift has also influenced the prevention and treatment of oral diseases, given the growing public interest in online information about dental caries symptoms and therapies.^3,4^ However, the low-quality information available on the internet about dental caries may lead people to adopt inadequate prevention and treatment methods, consequently contributing to the worsening of dental problems.^2,5–7^ For instance, people tend to discredit the role of sugar consumption in the development of caries lesions, while mistakenly believing in the importance of antibiotics or underestimating the preventive role of fluoride.^4,8^ These behaviors may have been exacerbated by the COVID-19 pandemic and political polarization, both of which contributed to the spread of health misinformation on social media.^9^ Social isolation further prompted individuals to seek information about oral health, alternative treatments, and medications on social media platforms.^7,10^

Dental caries is the most prevalent chronic oral disease worldwide, significantly affecting the quality of life of many individuals.^11^ In Brazil, it is the leading cause of toothache and tooth loss, and ranks among the costliest oral conditions to treat.^3^ Consequently, providing accurate and specialized materials to educate and advise the public is crucial to preventing the spread of inaccurate concepts and harmful beliefs among internet users. ^3,4,6,8^

In this context, infodemiology research is essential for detecting and monitoring oral health misinformation on social media, particularly in developing countries with significant socioeconomic disparities.^12,13^ Currently, Instagram has over 1 billion monthly active users worldwide,^14^ and represents the fastest-growing social media platform in Brazil. Therefore, this study aimed to identify misinformation in dental caries-related posts on Instagram in Portuguese and to analyze the factors associated with their sharing and engagement metrics.

## Methods

The authors followed the STROBE (Strengthening the Reporting of Observational Studies in Epidemiology) recommendations to ensure the quality and transparency of the study.^15^

### Study design

**Figure 1:**
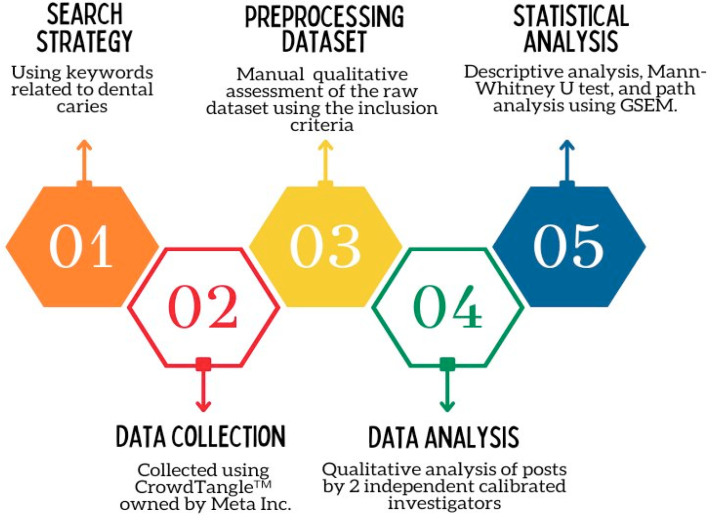
Schematic representation of the study design.

This longitudinal and retrospective infodemiology study characterized Brazilian Portuguese Instagram posts related to dental caries, as summarized in Figure 1. Initially, 500 posts were screened and collected using CrowdTangle™ (Meta Inc.), with data extracted on publication time, total interactions, and overperformance scores. Subsequently, two independent investigators conducted a content analysis of the posts, categorizing them based on author profile, content, motivation, facticity, format, and sentiment. Additionally, topic modeling analysis was performed using QDA Miner software with the WordStat module to identify underlying themes in both dental caries-related information and misinformation. The normality and homogeneity of the data were assessed using the Shapiro–Wilk and Levene tests, respectively. Given the non-parametric nature of the data, the Mann–Whitney U test was employed to identify significant differences in continuous variables. Path analysis using generalized structural equation modeling (GSEM) was then conducted to evaluate relationships between variables. Further methodological details are provided below.

### Ethics

This study did not require approval from the Institutional Ethics Committee of the Human Research Ethics Board at the University of São Paulo, seeing that it utilized publicly available data from the Internet and did not involve direct interaction with individuals.^13^ The de-identified raw data are shared on the Figshare repository.

### Search strategy, data collection, and preprocessing dataset

**Figure 2:**
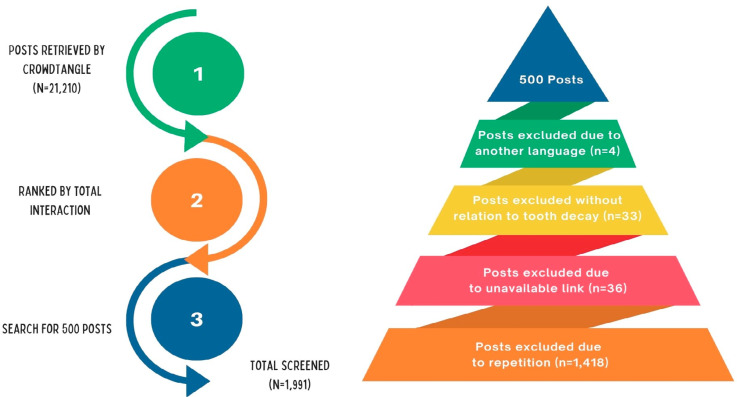
Flowchart depicting the selection process of posts.

The collection of posts related to dental caries on Instagram was conducted using CrowdTangle^TM^. The search strategy was constructed to enhance the retrieval of relevant data, based on an exploratory analysis of keywords. On October 26, 2022, a dataset containing 21,210 posts published between August 2018 and August 2022 was downloaded in .csv format using the following search terms: ["cárie dentária," "cárie," "caries," "lesão cariosa," "dente cariado," "lesões cariosas," "lesão de cárie"]. This dataset included the post ID, date and time of publication, profile information (name and country), format, content, total interaction (the sum of likes, comments, and views on a post), and overperforming score. Posts were ranked according to their total interaction. The overperforming score was calculated by dividing a post's actual interaction by its expected interaction, which is based on the number of followers of the author's profile. A positive score indicates that the post is performing well, thereby reaching a wider audience than the author's typical reach, whereas a negative score indicates underperformance. CrowdTangle's algorithm establishes benchmarks by analyzing the last 100 posts from an account, excluding the top and bottom 25%, and calculating the average number of interactions for the remaining posts across various time intervals (e.g., 15 minutes, 60 minutes, 5 hours). When a new post is published, the platform compares its metrics to this average and adjusts the score according to the weights assigned in each dashboard.^7,16^ To select the 500 most relevant posts based on total interaction, two independent investigators conducted a manual assessment, ensuring that only posts meeting the inclusion criteria were selected. This process resulted in the analysis of 1,991 Portuguese-language posts.^17^ Thirty-three posts unrelated to dental caries, four posts from non-native Portuguese-speaking countries, 36 posts with inaccessible links, and 1,418 duplicate posts were excluded, as shown in Figure 2. The selected posts were numbered and arranged sequentially in Google Slides (Google, Mountain View, USA), then converted to PDF format to facilitate consistent ethical analysis by different researchers. This systematic procedure allowed for independent analysis of the posts by different researchers at different times. Moreover, the topic modeling analysis required additional text preprocessing, which involved removing non-textual elements, punctuation, URLs, and stopwords. This process was conducted using QDA Miner software with the WordStat module (Provalis Research, Montreal, Canada).

### Data analysis

#### Qualitative analysis

Two independent investigators received comprehensive training to conduct a critical analysis of the content, including in-depth discussions of the key elements for characterizing the posts. Precise calibration was a priority for the study. Categorization was guided by predefined criteria, including author's profile (regular users, companies, dental offices, or healthcare professionals), motivation (financial or non-financial), sentiment (positive, neutral, or negative), facticity (whether the posts contained reliable information or misinformation), content (prevention or treatment), and format (photo, album, link, or video). This calibration process involved classifying 50 randomly selected posts from the sample and continued until the inter-examiner agreement reached an adequate level, defined as an Intraclass Correlation Coefficient (ICC) greater than 0.7. ICC values for all parameters ranged from 0.84 to 0.96, demonstrating a high degree of reliability. This rigorous training and calibration procedure was essential to ensure the accuracy and consistency of the investigators’ content assessments, following methodologies used in previous studies.^4,7,18^ In cases of disagreement, the researchers jointly reviewed the posts to achieve consensus.

#### Author's profile

Analysis of the author's profile considered the descriptions of Instagram profiles and pages, classifying them as regular users (including digital influencers or blogs), companies (commercial entities, stores, or profiles affiliated with media organizations or news agencies), and dental offices (dentists, healthcare professionals, clinics, or hospitals).

#### Motivation

Determining the author's intent in a publication poses a challenge in information analysis, given the difficulty in identifying underlying motivations, the influence of researchers’ subjectivity, and the complexity of distinguishing between honest and dishonest errors, in accordance with Poe's Law.^19^ In line with this principle, Wardle and Derakhshan^20^ proposed that the underlying motivation behind the creation of false or misleading content may stem from social interests (associated with specific groups), economic interests (aiming to profit through misleading information), political interests (influencing opinions aligned with political positions), or psychological factors (rooted in the pursuit of recognition or reaffirmation of pre-existing ideas). In this study, the motivation was categorized based on the perceived interpretation of the message, classified as financial or non-financial.

#### Sentiment

To reduce subjectivity, sentiment classification was based on visible indicators, such as smiles, happy emojis, or elements expressing positive feelings, as well as motivational messages and terms associated with the relief of painful symptoms or disease cure. Negative sentiment detection was based on the identification of expressions of sadness, texts with negative connotations, references to illness, pain, suffering, or emojis with negative connotations. Content presented objectively—without predominantly positive or negative emotional elements—was classified as neutral. This category encompassed scientific information and factual data, adopting a balanced, impartial approach, without inducing specific feelings in the target audience.

#### Facticity

The term "misinformation" was used to encompass false or misleading content, with or without the intention to cause harm. Thus, it incorporates both types of information disorders: misinformation per se and disinformation.^20,21^ The facticity of the content was evaluated against current scientific evidence, including guidelines, consensus statements, and systematic reviews related to dental caries management.^22–24^ A post was considered to contain misinformation only if it unequivocally presented false or misleading information with the potential to harm Instagram users.

#### Content

The content of the posts was categorized based on the perceived intention either to influence risk factors associated with dental caries (prevention)—such as high sucrose intake and dental biofilm formation—or to mitigate its clinical consequences (treatment), including caries lesions and pain manifestation.

#### Format

Each post was classified into a single format. If a post included a video, it was categorized as a video format, even if other media forms were included. The same classification method was applied to posts containing links. Consequently, posts categorized as photos did not include links or videos.

#### Topic modeling

Topic modeling analysis was conducted using QDA Miner software and automated text mining with WordStat. The model was based on factor analysis with Varimax rotation, enabling the identification of distinct topics across the datasets. This approach allowed a single word to be associated with multiple factors, capturing its use across different semantic contexts. Terms occurring fewer than 30 times were excluded to ensure the stability and interpretability of the factor solution. The thematic relevance of each topic was assessed based on two indicators: coherence, which measures the internal consistency and semantic similarity of the terms grouped within each topic, and eigenvalue, which reflects the proportion of variance explained by each factor, thereby allowing the prioritization of topics according to their representativeness within the dataset.

### Statistical analysis

Statistical analysis was performed using Stata SE 17.0 software (StataCorp., College Station, USA). Initially, variables were transformed into dichotomous categories based on the following criteria: author's profile (regular user or business/healthcare professional), motivation (non-financial or financial), facticity (information or misinformation), sentiment (negative/neutral or positive), content (non-commercial or commercial), and format (photo/album or link/video). Continuous variables—publication time, total interaction, and overperforming score—were dichotomized according to their median values. Dental practices, news agencies, and business profiles were grouped due to their similar financial histories. Previous evidence linking positive sentiment to increased engagement on social media guided the dichotomization of this classification.^25,26^ Since a post presents only one predominant sentiment (positive, neutral, or negative), neutral and negative sentiments were dichotomized in the same group to test the influence of positive sentiment on user engagement with dental caries-related posts.

The normality and homogeneity of the data were assessed using the Kolmogorov–Smirnov and Levene tests, respectively. Since the data did not follow a normal distribution, the Mann–Whitney U test was applied to compare total interaction and overperforming scores between the two categories within each dichotomized variable. Path analysis using GSEM was then employed to assess the associations between the dichotomized categories described earlier, based on the Theory of Planned Behavior.^27^

In this model, the ‘author's profile’ represented individual characteristics, ‘motivation’ represented intention, and the ‘sentiment,’ ‘content,’ ‘format,’ and ‘facticity’ of posts represented behavior. Since the volitional behavior (the published post) was already consummated, the constructs ‘attitude’ and ‘perceived behavioral control’ were equated to ‘favorable evaluation of the behavior’ and ‘perception of the ease of performing the behavior,’ respectively.

Environmental characteristics and injunctive and descriptive norms were represented by the internal latent variables ‘social environment’ (differences in societies) and ‘normative beliefs’ (differences in the sense of cohesion), considering that these influences could not be adequately measured due to a lack of variability resulting from the exclusive inclusion of posts in Portuguese.

**Figure 3:**
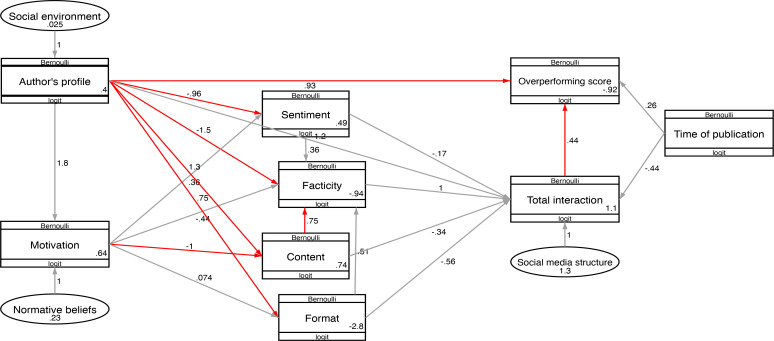
Path analysis generated by generalized structural equation modeling.

Interaction metrics were also incorporated into the model as behavioral outcomes, considering that the characteristics of posts can attract audiences at specific times. The internal latent variable ‘social media structure’ was included in the model, given that processes within social media—such as algorithmic sorting of searches and feeds or artificial interaction mechanisms—can influence total interaction. Additionally, the overperforming score varies according to audience interactions with posts from the same author, influenced by the time frame affecting trends in the total number of followers and posts over time (as shown in Figure 3).^16^ A significance level of p < 0.05 was adopted for all analyses.

## Results

Table 1 depicts a predominance of posts shared by business/health profiles (59.8%) with financial motivation (80.6%), expressing positive sentiment (54.8%), containing information (78.2%), concerning the treatment of dental caries (65.6%), and presented in the format of a photo/album (90.4%). The comparison of medians indicated that more recent dental caries-related Instagram posts (p = 0.004), posts shared by regular user profiles (p < 0.001), posts containing misinformation (p < 0.001), and posts addressing dental caries prevention (p = 0.005) presented significantly higher total interaction compared with their counterparts. Conversely, posts shared by business/health profiles (p < 0.001), posts motivated by financial interests (p = 0.007), and posts concerning the treatment of dental caries (p = 0.008) exhibited significantly higher overperforming scores compared with their counterparts (Table 2).

**Table 1:** Comparison of medians (q25, q75) for total interactions and overperforming score across time of publication, author's profile, motivation, sentiment, facticity, content, and format categories.

Variables		n (%)	Total interaction		Overperforming score	
			Median (q25, q75)	p-value	Median (q25, q75)	p-value
Time of publication						
	≤ 685 days	250 (50.0)	3716 (2737, 7083)	0.004*	1.75 (0.24, 3.95)	0.358
	> 685 days	250 (50.0)	3266 (2658, 4984)		2.15 (0.28, 3.91)	
Author's profile						
	Regular users	201 (40.2)	4984 (2825, 8697)	< 0.001*	1.48 (−1.06, 3.32)	< 0.001*
	Business/Health	299 (59.8)	3104 (2646, 4231)		2.44 (1.08, 4.44)	
Motivation						
	Non-financial	97 (19.4)	3812 (2769, 8195)	0.065	1.35 (−0.12, 2.89)	0.007*
	Financial	403 (80.6)	3378 (2658, 5744)		2.18 (0.32, 4.17)	
Sentiment						
	Negative/Neutral	226 (45.2)	3231 (2686, 5227)	0.052	2.30 (0.77, 3.77)	0.276
	Positive	274 (54.8)	3536 (2718, 6345)		1.73 (0.06, 4.07)	
Facticity						
	Information	391 (78.2)	3274 (2664, 5408)	< 0.001*	2.09 (0.48, 4.04)	0.070
	Misinformation	109 (21.8)	4654 (2961, 8697)		1.71 (−1.01, 3.38)	
Content						
	Prevention	172 (34.4)	3902.5 (2751, 8363)	0.005*	1.56 (−0.15, 3.46)	0.008*
	Treatment	328 (65.6)	3322.5 (2676.5, 5186.5)		2.31 (0.74, 4.25)	
Format						
	Photo/Album	452 (90.4)	3541.5 (2717.5, 6019.5)	0.076	1.95 (0.28, 3.92)	0.898
	Link/Video	48 (9.6)	3130.5 (2603.5, 4293)		2.12 (0.06, 5.29)	

(*) Asterisks indicate statistically significant differences between dichotomized categories (Mann–Whitney U test, p < 0.05).

**Table 2:** Associations observed in path analysis by generalized structural equation modeling (GSEM).

Variables		OR	SE	p-value	95% CI	
					LCI	UCI
Author's profile (Business/Health)						
	Social environment (L1)	2.72	(constrained)			
Motivation (Financial)						
	Author's profile (Business/Health)	6.09	105.73	0.917	1.04e-14	3.58e+15
	Normative beliefs (L2)	2.72	(constrained)			
Sentiment (Positive)						
	Author's profile (Business/Health)	0.38	0.08	< 0.001*	0.26	0.57
	Motivation (Financial)	1.43	0.36	0.150	0.88	2.34
Facticity (Misinformation)						
	Author's profile (Business/Health)	0.22	0.06	< 0.001*	0.13	0.37
	Motivation (Financial)	0.64	0.18	0.106	0.37	1.10
	Sentiment (Positive)	1.44	0.37	0.158	0.87	2.38
	Content (Treatment)	2.11	0.59	0.007*	1.22	3.64
	Format (Photo/Album)	1.66	0.64	0.187	0.78	3.53
Content (Treatment)						
	Authors' profile (Business/Health)	3.69	0.78	< 0.001*	2.43	5.59
	Motivation (Financial)	0.36	0.01	< 0.001*	0.21	0.63
Format (Photo/Album)						
	Author's profile (Business/Health)	2.11	0.78	0.040*	1.03	4.33
	Motivation (Financial)	1.08	0.49	0.870	0.45	2.60
Overperforming score (> 1.95)						
	Author's profile (Business/Health)	2.54	0.50	< 0.001*	1.72	3.75
	Total interaction (> 3477)	1.56	0.30	0.023*	1.06	2.28
	Time of publication (> 685 days)	1.29	0.24	0.162	0.90	1.86
Total interaction (> 3477)						
	Author's profile (Business/Health)	0.31	0.49	0.463	0.01	7.25
	Facticity (Misinformation)	2.73	3.69	0.458	0.19	38.71
	Sentiment (Positive)	0.84	0.29	0.621	0.43	1.66
	Content (Treatment)	0.71	0.36	0.505	0.27	1.92
	Format	0.57	0.48	0.502	0.11	2.95
	Time of publication (> 685 days)	0.65	0.40	0.483	0.19	2.18
	Social media structure (L3)	2.72	(constrained)			

(*) Asterisks indicate statistically significant associations between categories (p < 0.05).

The GSEM analysis identified eight significant associations among the categorized variables. Positive sentiment was negatively associated with business/health profiles (OR = 0.38; p < 0.001). Misinformation was negatively associated with business/dental health profiles (OR = 0.22; p < 0.001) and positively associated with posts about the treatment of dental caries (OR = 2.11; p = 0.007). Additionally, treatment of dental caries was positively associated with business/health profiles (OR = 3.69; p < 0.001) and negatively associated with financial motivation (OR = 0.36; p < 0.001). Furthermore, photo/album format was positively associated with business/health profiles (OR = 2.11; p = 0.040). Meanwhile, the overperforming score was positively associated with business/dental health profiles (OR = 2.54; p < 0.001) and with higher total interaction (OR = 1.56; p = 0.023). Model fit was assessed through postestimation indices, namely the Akaike information criterion (AIC = 4,522.40) and Bayesian information criterion (BIC = 4,657.27.

In addition to the quantitative findings, topic modeling analysis provided valuable insights into the thematic structure of the content. Considering only results with an eigenvalue greater than 1 among posts containing misinformation, four dominant topics were identified: (a) dental caries treatment, (b) diet, (c) oral hygiene, and (d) microorganisms (Table 3). Conversely, posts containing accurate information revealed three main topics: (a) dental caries prevention, (b) oral hygiene, and (c) dental caries treatment (Table 4).

**Table 3:** Topic modeling of Instagram posts containing dental caries-related misinformation published in Brazilian Portuguese.

Topic	Significant words	Coherence (NPMI)	Eigenvalue
Dental caries treatment	Dentistry; Love dentistry; restorative dentistry; caries; dentistry; pediatric dentistry; dentist; gum.	0.465	3.810
Diet	Childhood; healthy; help; nutrition; health; development; disease; problems; oral; caries; dental.	0.387	2.600
Oral hygiene	Floss; dental; use; brushing; tooth; oral; toothbrush; fluoride; toothpaste; important; hygiene; brush; plaque; health; amount.	0.360	1.940
Microorganisms	Bacteria; caries; breath; plaque; tartar; mouth; problems; cause; enamel; tooth.	0.341	1.730

**Table 4:** Topic modeling of Instagram posts containing dental caries-related information published in Brazilian Portuguese.

Topic	Significant words	Coherence (NPMI)	Eigenvalue
Dental caries prevention	Prevent; diseases; help; nutrition; childhood; prevents; healthy; problem; development; let's; dental; health; caries.	0.611	4.370
Oral hygiene	Hygiene; lesions; tooth; caries; mouth; patient; diet; habit; years; child; feeding; oral; baby; milk; large; brush; clinic; disease.	0.347	2.420
Dental caries treatment	Caries; dentistry; pediatric dentistry; odontology; tooth.	0.308	2.510

## Discussion

This study offers a comprehensive analysis of dental caries-related information shared in Portuguese on Instagram, focusing on metrics that assess misinformation and user engagement. The findings indicate that 21.8% of the analyzed posts contained misinformation, primarily associated with content shared by regular users regarding dental caries treatment. No significant relationship was observed between misinformation and user engagement; however, the median total interaction was significantly higher among posts containing misinformation than among those with accurate information. Overperformance scores were positively correlated with business and health-related profiles, as well as with higher total interaction levels. Financial motivation was less associated with treatment-related posts, reflecting the tendency of promotional content to focus on preventive technologies. Lastly, while the misinformation predominantly addressed dental caries treatment, diet, microorganisms, and oral hygiene, the accurate information mainly covered prevention, treatment, and oral hygiene.

Business and health professional profiles were associated with lower levels of misinformation, confirming their potential as more reliable sources of information for promoting digital health education. Previous research demonstrated that young adults who viewed educational Instagram posts by healthcare experts reported higher credibility and trust in the messages, a finding explained by the concept known as the *expertise heuristic*. This heuristic refers to a mental shortcut in which individuals judge information as more credible when it originates from someone perceived as an expert. Thus, health professional profiles are considered more reliable sources for dental content than friends or social media influencers, aiding in the dissemination of trustworthy information.^28^ These results indicate a strong association between post performance and profile characteristics. Business and health professional profiles appeared more successful in engaging the audience. Moreover, profiles with higher overperforming scores also exhibited higher total interaction. Companies, dental offices, and media outlets often tailor their messages to attract clients on Instagram, sometimes paying to promote their content, which not only increases engagement but also enhances information dissemination.^19^

The data analysis also revealed that more recent posts shared by regular users, particularly those about caries prevention and containing misinformation, paradoxically showed higher engagement metrics. This trend contrasts with previous studies,^7,18^ indicating increased user interest in content related to dental caries. Sensationalized misinformation about prevention—such as claims that "guava seeds grow inside a decayed tooth" or "bacteria causing dental caries and tuberculosis can be killed by ingesting cashew seed"—can pique curiosity, encouraging interactions such as shares and comments. Users uncertain about the accuracy of information are more likely to engage with sensationalized misinformation.^29^ Therefore, it is not surprising that many opt for sensationalism over accuracy to attract greater attention.^30^

Promotional health content might stand out in the digital landscape despite being less emotionally engaging. The association between misinformation and posts about dental caries treatment demonstrates the existence of misleading dental practices disseminated on the Internet. This study identified content promoting therapies such as the use of herbs, cashew seeds, and coconut oil for the prevention and treatment of dental caries, aligning with findings from a study conducted on YouTube.^31^ However, some of these methods may pose risks to oral health and undermine patients’ trust in healthcare professionals.^1^ With the expansion of the internet, alternative health treatments have gained popularity, driven by dissatisfaction with conventional medicine and a rejection of science.^1^ Misinformation spreads faster than accurate information, making people more likely to believe it, particularly when it aligns with their confirmation bias.^8^ Social media platforms facilitate the dissemination of information about alternative treatments, often lacking robust scientific evidence regarding their effectiveness and safety.^1,7,19,31–33^

Furthermore, neutral and negative sentiments were predominantly associated with posts shared by business and health profiles, possibly because these profiles focus on disseminating information about disease etiology, pain, aesthetics, and suffering, with an emphasis on prevention strategies and professional treatments. A social media study revealed that posts with negative or neutral sentiments garnered more interactions, highlighting dental stress and afflictions, and fostering understanding among the target audience, particularly parents and caregivers seeking reassuring advice.^18^ This finding reinforces the negativity bias in online health communities, where negative content receives greater support.^23^

On the other hand, posts about the treatment of dental caries were less associated with financial motivation than preventive ones, indicating a prioritization of health information based on criteria beyond financial considerations. This may explain the popularity of alternative treatments for dental caries and the influence of socioeconomic context on decision-making related to dental treatment, pointing out disparities in gaining access to dental services in Brazil.^34,35^ It may also reflect the promotional strategies typically linked to technologies for preventing dental caries found on social media.

The topics identified in posts containing misinformation suggest a tendency for misleading content to adopt narratives that promote unproven therapies and treatments under the guise of conventional dental care, disseminate unfounded dietary recommendations—particularly within the context of pediatric health, where informational vulnerability is heightened—and misrepresent the role of microorganisms in oral health. In contrast, posts containing accurate information were aligned with evidence-based practices, emphasizing health promotion, risk factor management, and the implementation of scientifically supported preventive and therapeutic interventions.

This study presents some limitations. Firstly, it focused solely on posts about dental caries published in Portuguese, which may limit the understanding of cultural influences on oral health misinformation. Secondly, although the findings provide valuable insights into the factors driving the sharing and engagement of dental caries misinformation, they did not consider the demographic characteristics of users’ profiles, restricting the understanding of which population segments are more susceptible to or engaged with misinformation. Furthermore, the sample size was limited by the inherent challenges of content analysis, which required manual identification and classification of data. Nevertheless, this approach was grounded in methodologies from previous studies that used similar sample sizes.^4,7,19^

## Conclusion

This study revealed that dental caries-related Instagram posts frequently contain misinformation about treatment shared by regular users. Financial motivation was less associated with treatment-related posts, reflecting the promotional focus usually linked to technologies for disease prevention. Although these findings were not associated with higher user engagement, they underscore concerns regarding the dissemination of misleading information about dental issues online. Business and health professional profiles demonstrated good potential to disseminate reliable information with higher engagement metrics. While easy access to information can benefit public health interventions and improve digital literacy, the challenge of filtering accurate information about oral health may impair people's quality of life. Therefore, these results contribute to a better understanding of the factors influencing the sharing of misinformation on social media, and can facilitate the diffusion of more reliable oral health information across digital platforms.

## Data Availability

The authors declare that all data generated or analyzed during this study are included in this published article.
